# Sanitary Work in the State of New York1Address delivered at the seventh annual conference of the Sanitary Officers of the State of New York, held at Buffalo, October 16, 17, 18, 1907.

**Published:** 1907-12

**Authors:** Eugene H. Porter

**Affiliations:** Albany, State Commissioner of Health


### BUFFALO MEDICAL JOURNAL.

VOL. LXIII. DECEMBER, 1907. No. 5

### ORIGINAL COMMUNICATIONS.

### Sanitary Work in the State of New York.[1]

BY EUGENE H. PORTER, M. D., Albany,
State Commissioner of Health.

WE are gathered this afternoon to inaugurate the seventh annual conference of the Health Officers of the State of New York. This is a day of salutations. We are met here to greet a new sanitary year, to welcome those who have come to join our ranks and to exchange words of encouragement with one another. It is well that from time to time the principles that govern this conference, the traditions that affect it, the aspirations that move it, should court the fullest inquiry. For the conference is to a great measure representative of the department. That which moves the one, moves the other. If there is development and advancement here, there is, you may be sure, progress and fruitfulness there. But if our teaching has hardened into dogmatism, our precepts into formulas, and our system into routine, then is our custom a yoke, not of freedom but of bondage. We have fallen somewhat below our standards. For in our day we are restless, drawn by a discontent not always divine, dragged down by laxity, selfishness and the tremendous materialism of the age. And besides all this there remains the monumental words of the first and greatest physician of western civilisation "Life is short, and art long, occasion fleeting, experiment fallacious and judgment difficult."

But while the road is difficult and advance not easily won, knowledge is greater, conviction clearer, purpose more resolute and accomplishment nearer.

And so if our meeting here shall be a fresh incentive to continued labor, if it shall bear a particular and special message to each listening ear, and if in addition to that comradeship, so enjoyable, and that living sense of power through unity of organisation, so essential, it shall bring to each of us a more complete

1. Address delivered at the seventh annual conference of the Sanitary Officers of the State of New York, held at Buffalo, October 16, 17, 18, 1907.

realisation of the problems that confront us, a wider and keener mental vision, an inspiration leading to better public service, the purposes of this conference will have been fully attained.

This is the largest gathering of health officers ever held in this state or for that matter in any other state in the Union and that it is so is due to the untiring devotion to public duty and the constant enthusiastic interest in sanitary affairs always manifested by the health officers of the Empire State.

In greeting you this afternoon I find some difficulty in adequately expressing how much your presence here is appreciated and how fortunate we esteem ourselves that we have the oppoitunity to meet so many of our co-workers in sanitary matters.

The conference extends its heartiest greetings to our good friends the citizens of Buffalo. This splendid audience is an index of the public spirit and intelligence of the people of this municipality and in sanitary, as well as in other things, Buffalo is certainly a city of the first class.

### SOME SPECIAL DEPARTMENT WORK.

It has seemed to me that the best use of my brief period of time would be to tell you some of the things we have done since the last conference, and then discuss briefly a few important matters that closely concern next year's work. So much has been done, compared with anything we were able to undertake before, that I can hardly do more than mention some of the many lines of endeavor entered upon.

1. *The investigation of summer resorts* has been continued and a large number of hotels have been personally inspected. The reports secured by the blank forms sent out last year to the owners ot summer hotels and boarding-houses have been thoroughly studied and tabulated, and where the conditions are unsatisfactory, and possible or probable danger threatens visitors, measures have been taken to correct the evils. The number of summer resorts in this state is enormous, and to properly supervise their sanitary condition requires more money than is now available. I hope next year, however, to approximately complete this work and so prevent many cases of "Summer resort typhoid."

2. *Sanitary investigations of various cities.*—It is not enough to tabulate death returns. These melancholy statistics taken in bulk do indeed serve to remind us of "that bourne from which no traveler ever returns," but afford very little other information of practical value.

But when intelligently studied they are employed for purposes of comparison, and the results attained, interpreted by the sanitary methods of today, they may serve as beacon lights pointing out existing danger and demanding its avoidance.

So the dead shall speak to us and these voices from the grave may not be lightly disregarded. And when it was found that in one of the cities of this state the average annual death-rate from typhoid for ten years was 138, in another 87, in another 82, in still another that the mortality from tuberculosis was three times what it should be, and in still another city the conditions indicated by the tables pointed unmistakably to an epidemic outbreak of disease, it needed no labored argument to convince me that one of the urgent duties right before the department was the correction, if possible, of these conditions.

And so we have begun this summer a sanitary investigation of the conditions existing in various towns and municipalities in this state. In this study were included the water supply and all possible existing sources of contamination; the method of disposal of sewage; the method of garbage disposal, if any; the chief occupations of the people; the number and kind of manufacturing or other industrial establishments; the number and character of tenement-houses, together with many other matters bearing relation to the general investigation. Some of these investigations are completed, others are still under way.

When these reports are finally passed upon the department believes that it will be able to give to each of these municipalities specific and substantial aid in correcting the very serious existing dangers to health and life. It will not only point out the evil that exists but it hopes to be able to suggest the adequate remedy, and it will use all the influence and moral force at its command to effect a change for the better.

But the department does not undertake to do the required work. The town must work out its own sanitary salvation. The department investigates, studies conditions, suggests, gives expert advice, points out the way. The rest of it belongs unmistakably to people dwelling where the conditions exist. And this it would seem is one of the most important functions of your department of health: to give to any town or municipality needing it expert advice and aid in the avoidance of sanitary perils and their speedy correction if in existence. It will be readily seen that such work as this required much time and considerable expenditure of money. But we went as far as we could.

3. *The pollution of our streams and lakes* is one of the most important and difficult questions before us. It is obvious that a problem so serious, affecting so many and varied interests, must be dealt with in a broad and comprehensive fashion. As a basis for such treatment positive information is needed. It became, therefore, imperatively necessary to thoroughly study all the principal watersheds of this state. This work included the present pollution of streams, their suitability as sources of water supply, the wastes from factories and creameries, the refuse from pulp mills and many other important facts. Sanitary maps have been prepared of many of these watersheds on a scale to show plainly each stream of appreciable size, with the location of each source of water supply, and all important sources of pollution. The Oswego, Upper Hudson, Lower Hudson, Susquehanna, Genesee and Niagara watersheds are now practically completed.

4. *Examination of all public water supplies.*—It has always seemed plain to me that one of the most legitimate functions of the State Department of Health was a continuous and efficient supervision of public water supplies. The work done by Massachusetts in this line has been most excellent and far ahead of anything we have ever been able to do. But the increased appropriation in the supply bill rendered it possible to begin this important work and every public water supply in the state has been examined and reported on. These examinations will be repeated at regular and necessary intervals, and where the supply is contaminated the cause will be removed as promptly as possible. Hand in hand with this work goes the

5. *Investigation of all sewage disposal plants.*—The value of a sewage disposal plant depends upon its efficiency. The quality of the effluent tells the story. So every disposal plant has been examined and every effluent has been analysed. The results of these examinations will be used where indicated to better conditions.

6. *Examination of the eyes and ears of school children.*—Last year you will remember it was stated that the department had in mind the institution of an examination of the eyes, ears, noses and throats of school children. This, of course, was to be done with the coöperation of the Education Department.

I am very glad to announce to you that the plan proposed last year by Dr. Schenck, with some slight modifications, goes into operation this fall in 450 high schools of this state. As Dr. Schenck will tell you a little later just how all this is to be done, I will not go into details.

7. *Examinations of new series of meats.*—The report of the laboratory on the first series of samples of meat products excited much attention and had a most excellent effect. A second series of samples of meats has been collected and examined and the full report will be published in a short time.

8. *Analysis of beers, etc.*—A large number of analyses have also been made of beers, wines and whiskies which will shortly appear in the bulletin. No appropriation was made for these examinations, but it seemed necessary to make them and the expense has been paid from the general fund.

9. *Tuberculosis exhibition.*—Another line of special work undertaken this summer is the tuberculosis exhibition, the first ever owned by the state, and the hygienic laboratory exhibit which is but the nucleus it is hoped of a much larger collection. The work of preparing these exhibits has been most onerous, the more so because of limited means, but the enthusiasm of Dr. Pease and his faithful assistants never flagged. It is our purpose to further perfect these exhibits, particularly the tuberculosis exhibit, and use it as a traveling exhibit for educational purposes. It is one of the things most sorely needed by the department. Already we have received many requests from cities and towns to have it sent to them.

10. *Traveling libraries and bacteriological outfit.*—You will notice also in the exhibition here a collection of books and some apparatus for tuberculosis work. These are traveling outfits—the one a small collection of specially selected books on sanitation for the use of health officers, and the other a practical and always ready outfit for bacteriological work to be done on the spot where investigation and action are needed without delay. Although these things may not seem large, yet they are of no little importance because they will add materially to the efficiency of our work.

11. *Special circulars.*—The very general interest now manifested by the more intelligent citizens of our country on sanitary matters is due not so much to the general advancement of scientific knowledge, by the arduous labors of students and laboratory workers, as it has been to the dissemination of this increased knowledge. If these discoveries of truth never passed beyond the closed doors of our laboratories, or could only be found between the covers of technical pamphlets, their influence and power for good would be as nothing. They would be mummified truths—embalmed but not available. Education then must continue to be the watchword. Education in sanitation spells progress in sanitation, and no positive and decisive advance may be made until our fellow citizens are educated to the point where they are generally convinced that the advance must be made.

So we have determined to begin the publication of a series of circulars and pamphlets on topics vitally concerning the public health for popular distribution. Work on the first series has already begun, and the topics and dates of publication will be duly announced in the bulletin. It will be a very modest beginning, but we hope these little pamphlets will prove an important factor in our campaign of education.

What may be termed the first one of the series is the very complete and instructive prize essay by Dr. Knopf on the Treatment of Tuberculosis. This may be found at the registration office, and a copy will be given to each health officer attending the conference.

These then, my friends, are some of the things we have tried to do since our Syracuse meeting a year ago. Of course, in addition to these lines of work, the routine work of the department, which has quadrupled during the last two years, has been going steadily on.

### APPROPRIATIONS.

Efficiency and appropriations—the union of affinities, the one a compliment of the other—married by general consent—let no legislature put them asunder.

For it ought to be true, even if it is not altogether so as yet, that no appropriation of public moneys should be made without a definite expectation of efficient and honest public service in return. To the question: What is the expense of a certain line of work? must be added the significant inquiry "What service has been rendered for that expense?" But there can be no service without means: there can be only inadequate service with insufficient funds: there can be efficiency in its fullest sense when the appropriation is enough to meet actual needs.

The last legislature increased the appropriation for our department by almost $42,000. The Governor allowed every increase asked for by the department and stated that he did so in order to increase the efficiency. This increase of income has enabled the department, for the first time, to begin lines of work that could not be undertaken before because of the expense.

But the appropriation does not as yet, by any means, meet the requirements. The total amount of money available for the department, exclusive of the cancer laboratory, is still less than $100,000. We are still severely crippled in the amount allowed for "Investigations," for the "Division of Engineering," and for the "Division of Laboratory Work." I have no hesitation in stating that the efficiency of the department is materially lessened and its administration hampered by lack of funds. Pennsylvania for the two years beginning June 1, 1907, has appropriated for its Department of Health—after the payment of the annual salary of the Commissioner, $10,000, and the salaries of numerous other officers, the sum of $1,459,312—$400,000 of which is for "the dissemination of knowledge relating to the prevention and cure of tuberculosis." I may add that the appropriation made by Massachusetts also exceeds that of New York. All we want is enough to do thoroughly and well the work that lies before us.

### PURE FOOD LAW.

The attitude of the department regarding a pure food law was made so clear at the Syracuse conference that it seems unnecessary to dwell upon the subject now. It perhaps is sufficient to say that the department believes the state should have, without delay, a wise and practical pure food law, and that the enforcement of it should be placed in the hands of the health department.

### THE CANCER LABORATORY.

It is perhaps appropriate in the city of Buffalo, which contains an offshoot of the Laboratory Division of the State Department of Health, the Cancer Laboratory, for a few words to be said on the subject of this most serious and intractable disease. It might be well if we advised the citizens of the state not to put too much credence in the newspaper reports as to the curability or incurability of cancer.

The laboratory here has been working faithfully and well on the problem of determining a probable cause for the disease. It has not concerned itself with attempting to find a cure. Its work, therefore, may not be popular, but it is scientific, and in the end will accomplish more than would a search first in this direction and then in that for possible remedial agents. When we have determined the cause of the malady there is little doubt that a remedy will be forthcoming comparatively soon after. It is true that deaths from cancer are increasing, and the situation is a serious one, and the state has a right to impress its seriousness upon the laboratory here, and urge that the money it appropriates shall show results.

In addition to exploiting various cancer cures, some newspapers have done considerable harm by publishing alarming articles on the contagiousness of cancer. Unfortunately the origin of those is to be found among some irresponsible members of the medical profession, who, not realising the serious nature of their claims, and their far-reaching effects upon the general public, have drawn hasty conclusions from insufficient data, and have published in the medical press their immature opinions, more than suggesting the contagiousness of the disease. Such an announcement is eagerly seized upon by the sensational press, and the public, already educated by the physicians and sanitarians to accept specific germs as the cause of cancer, and to recognise the infectious character of germ diseases, have proved fertile soil for the spread of the idea that cancer is contagious. We need to let the public know that there is no sufficient evidence as yet that this is the case.

PUBLIC HEALTH DIPLOMA

In the spring of this year I received from the secretary of the Medical Society of the State of New York, a copy of a resolution passed by the society at its last annual meeting. The import of this resolution was that it was the opinion of the society that only those physicians should be appointed as health officers who could show evidence of special training in public health work. I want to say that the department is in hearty sympathy with the spirit of this resolution, and is very glad to recognise the interest manifested by the medical profession in this state in matters of public health. There are, however, practical difficulties which must be taken into consideration.

I take it that those who framed the resolution, and were responsible for its passage by the society, had it in mind to secure in this state the adoption of the method in vogue in Great Britain and other European countries, in appointing medical officers of health. Let us consider for a moment the requirements as they exist in Great Britain.

A candidate for appointment as a medical officer of health must be possessed of special qualifications, known briefly as the D.P.H., which stands for a diploma in public health. The examination for this diploma consists of two parts. Before the candidate can be admitted to part one of the examination, he must be 23 years of age, and have been a qualified practitioner of medicine 12 months, and after his graduation he must have had six months practical instruction in hygienic chemistry, bacteriology, and the pathology of the diseases of animals transmissible to man. This part of the examination covers the following subjects: (1) Physics in their application to health, and with reference to ventilation and heating; water supply and sewerage. (2) Chemistry in its relation to air, water, food, soil, and sewage. (3) Microscopical examination of air, water, food, articles of clothing, and parasites. (4) Bacteriology in relation to sanitary work.

Before admission to the second part of the examination, the candidate must show that after his graduation he has spent six months in acquiring practical instruction in the duties, routine and special, of public health administration under a medical officer of health in a community of not less than 30,000, and also has had three months' experience in a hospital for infectious diseases. The subjects embraced in part two of the examination are as follows: (1) Origin, pathology and prevention of diseases, with special relation to infectious diseases. (2) Effects of unwholesome air, water and food. (3) Diseases of animals in relation to the health of man. (4) Influence of occupation---unhealthy trades. (5) Influence of climate. (6) Sanitary administration in relation to requirements of houses and other buildings; sanitary engineering. (7) Construction, arrangement and management of hospitals. (8) Statistics in relation to health. (9) Sanitary laws, including by-laws, orders and regulations. (10) Duties of sanitary officers.

That every health officer should be possessed with the knowledge indicated in the above outline is a consummation devoutly to be wished. But under the present circumstances it cannot be expected. In the first place, an investigation conducted by the department, has revealed the fact that there is not an institution in this state offering a course of post-graduate instruction covering these subjects. The prospectuses of the various medical schools show that hygiene and sanitary science are taught to students in their first or second years. The scope of the instruction is not as full as is necessary for the mental equipment of the health officer, if we are to put British standards in force; and, moreover, we all know from practical experience that the subjects taught in the first two years of a medical student's career are considered by him as very largely theoretical, and therefore of not sufficient practical importance for him to make really a part of his medical knowledge. If he retain sufficient to answer satisfactorily the few questions set by the State Board of Examiners he considers he is doing well.

Again, if opportunity were afforded for post-graduate instruction of the character I have outlined, it is a very serious question whether the recompense received by the majority of health officers would warrant them in going to the expense of obtaining a special training. I want to say, then, to the medical profession in this state, that these two practical obstacles must be removed before the department can adopt for its own the rule suggested by the state medical society.

As to the first obstacle, the department intends to do what it can to provide some opportunity for instruction in sanitary science and public health. During the past year sanitary institutes have been held by the department in various parts of the state, which have afforded opportunity for the acquirement of increased knowledge on the part of the health officers. But we recognise that we have only scratched the surface. The Division of Publicity and Education, however, is seriously considering the problem, and I hope to be able a year from now to make an announcement at our next annual conference of plans for some special courses of instruction for health officers, and those desiring to become such. If the department accomplishes this, the medical profession must do its part, and I believe this part can best be the removal of the other obstacle to which I have referred. The organised medical profession in this country has recently shown its power in the matter of fixing the recompense for services rendered, by forcing some of the largest life insurance companies in this country to restore the fee which was granted for examinations prior to the recent enforced retrenchment in insurance circles. I want to suggest that this same power of the organised medical profession can well and rightly be employed in securing adequate recompense for the health officers throughout the state. With very few exceptions these public servants are miserably underpaid. They are the means of saving millions of dollars every year, and a fair premium on this saving would furnish adequate compensation for these faithful public servants. I would like to see the medical society of this state initiate a movement of reform in this matter, and I hope to learn by our next meeting that the subject has been well discussed in many medical circles.

My answer, therefore, to the Medical Society of the State of New York, addressing me on the subject of requiring evidence of special qualifications from candidates for appointment as health officers, is, that the department is willing and anxious to comply with the suggestion as soon as it is feasible to do so.

### TYPHOID AND POLLUTION

A fact most gratifying to record is that, during the past two years, marked and increasing progress has been made in lessening the pollution of our rivers, streams and lakes. That public sentiment is more and more actively supporting the position of the department in this important duty is unquestionably due to the general dissemination of knowledge concerning the dangers of unrestrained pollution. It is a striking illustration of the fact that you must first educate and then advance. In some cases you educate by advancing. But in this matter, so far-reaching were its consequences, so many important interests, both public and private, were vitally interested, so many difficulties and peculiar embarrassments were in the way, that it was essential that the people should see as clearly as possible the imperative and urgent necessity for this sanitary reform before much progress could be made. That there is now throughout the state a general sentiment that our waters must be purified there can be but little doubt; when occasion arises it must be crystalised, specially informed, and wisely directed.

It is needless now and here to again enter upon an elaborate discussion of the dangers of polluted and infected waters. If we estimate that 20,000 cases of typhoid occur in New York state in one year with 2,000 deaths, the money loss to the state is easily figured. Allowing each life to be worth $3,000, a low estimate, as the young and vigorous are most often victims, and estimating that 15,000 of the 20,000 cases were men and were kept from labor 40 days, and putting the value of a day's work at $1.50, the total pecuniary loss to the state amounts to $7,000,000. We know without telling what misery and suffering were caused by these thousands of cases, what grief and distraction occurred when death entered hundreds of homes; and to this must be added the very serious business loss of a community when the disease occurs in any way approaching epidemic form. Let us remember also the danger of infection by tuberculosis,—a danger more serious and grave, I fear, than it is at present supposed to be.

Then, too, when the stream is not infected, the conditions of pollution existing cause a disfigurement of its natural features often peculiarly repulsive. The banks are covered with deposits offensive to both sight and smell; dirty and slimy scum floats on the surface of the stream and accumulates in eddies and coves; the bed of the river is covered with a viscid material that rises promptly when disturbed and yields to the grieved nostril a pungent odor peculiar to itself. Fishing is impossible for there are no fish,—boating as a pleasure is destroyed, the banks of the river no longer yield enjoyment. The comfort and enjoyment of the people living along such a stream are impaired and sometimes totally destroyed.

We are all agreed that such conditions should not continue to obtain. The policy of the department remains as announced at Albany two years ago; that the continued pollution of our waters must cease. It does not expect this pollution will cease in a day, nor in a year, but it does expect that within a comparatively short period it will be entirely stopped. When a given case of water pollution presents itself for consideration two questions arise which must be definitely answered before advance can be made. The first question is what is the best and wisest thing to do in order to correct the existing conditions? and the second question is, How is it to be done? The first question the department will always try to intelligently and comprehensively answer. To know what to do is perhaps the most important part in purifying our waters and the department feels it is a clear duty to furnish that information if possible.

The answer to the second question will, it is hoped, correct a great amount of misapprehension as to the powers and duties of the state health department under the existing law. It is evidently not the present intention of the state to remove from localities or the individual citizens inhabiting such localities, certain duties and responsibilties relating to health matters. Their powers begin in most instances where those of the commissioner of health end, and under the term of the present health laws the reply to the second question must be—it must be done by the town or individual affected. In other words, in the majority of cases when the department is appealed to for relief from water pollution or abatement of nuisances, the department may point out through its experts, the proper way to correct these evils; but the town or individual must do the rest. If the town above, on the stream, is polluting the water seriously, the town below must take the active and legal steps in the majority of cases to have the nuisance abated. The department may investigate, declare the existing conditions an intolerable nuisance and report its conclusions, including the remedy for the evil, to both towns. But in the majority of cases that is as far as it can now go.

The responsibility rests where it ought to rest in many cases, on the shoulders of the local authorities, who are much too willing very often to shirk their plain duty. It is as much the duty of health boards to enforce the health laws of this state as it is that of the commissioner of health, and the local authorities of the towns of this state should shoulder their responsibilities and not attempt to place them elsewhere. The department has no arbitrary powers of the matter of stream pollution. It cannot in the majority of cases, directly order a town to take its sewage out of a stream or direct it preëmptorily to construct a sewage disposal plant. It can, of course, through the Attorney-General, appeal to the courts, but so can the towns and individuals in the majority of cases, and it is the intention of the law as it stands that they shall do so. I am not discussing the value or effectiveness of the present health laws. I am simply trying to make clear their practical working. In a number of cases the department has no doubt been deemed very indifferent and ineffectual when it has done all the law permitted and stopped only because the limitations of the law compelled it to.

### TUBERCULOSIS.

That tuberculosis was both communicable and preventable was as well known to sanitarians twenty years ago as it is today. And it may be taken as a most striking illustration of the slowness with which knowledge filters down from the laboratory and the school to the profession and the public, that not until comparatively recently has such a realisation of conditions obtained as to cause an imperative demand for the enforcement of preventive measures. It may be safely said now, I think, that it is an universally accepted fact that some kind of supervision is necessary, opinions only differing as to the extent of the supervision and the manner of its enforcement.

You will undoubtedly agree that there are at present some thousands of our fellow-citisens throughout the state in the early stages of tuberculosis who might, under favorable conditions, be kept among the living. I am not speaking now of advanced cases, who must inevitably be soon numbered with the dead, nor of those incipient cases receiving competent care, but of those who are entitled to treatment which they do not receive. Shall the state that houses, treats and cares for both the acute and chronic insane refuse to consider the needs of those afflicted with tuberculosis? Are the blind, the crippled and the epileptic to be given state aid and the consumptive refused it? And it must not be forgotten the indigent consumptive in any stage of the disease, unlike the wards of the state just mentioned, is a very constant menace and danger to those with whom he is living. If we admit then, that the state should take some action, the instant question arising is, What action?

It is indeed a most grave and difficult question and a complete and thoroughly satisfactory answer is perhaps impossible to give at present, nor would I attempt at this conference to suggest even a tenative policy for our state were it not for the overshadowing importance of the subject and a clear conviction that some kind of action should be taken as quickly as possible.

You will remember Pennsylvania has an appropriation of $400,000 for the dissemination of information regarding tuberculosis, its prevention and cure. Our department has in the last supply bill an allowance of one thousand dollars for the creation of a traveling tuberculosis exhibit. There will be practically no money left when that exhibit is fully completed. But if we go ahead with the courage of our convictions and demonstrate the efficiency of our plans, the money to carry on the work will come. In this campaign then, against tuberculosis, the following steps might be taken.

1. *Notification and Registration.*—Without notification it is apparent that any real progress is rendered impossible. You cannot give aid to any consumptive until you know where he is, nor can it be known what help he requires until the case is properly reported and the conditions are described. Every physician in the state should promptly notify his health officer of any case of tuberculosis occurring in his practice, using for that purpose the cards and blanks already provided by the department. The health officer in turn reports all cases to the department. There is no publicity and all such returns are strictly confidenital communications. This step was begun on January 1 of this year, and while the registration of cases has been fairly good, the step is as yet only half taken. To have it entirely satisfactory we must have the active and cordial support of the profession at large and more systematic work from some of our health officers.

2. *The establishment of district station.*—The medical officers of the department have been for some little time so selected that it is now possible to divide the state into districts, each one having within its boundaries at least one medical officer.

At some central point in this district a station may be established in charge of the medical officer and should contain a supply of diphtheria and tetanus antitoxin, a bacteriological outfit, report cards and blanks, and a full supply of all the circulars and pamphlets issued by the department on tuberculosis. The station would also contain outfits for the collection of sputum in order to facilitate an early and definite diagnosis. The station and its contents would be an educational center. The circulars would be for both physicians and the laity and their distribution would be made by the medical officer and the health officers in the district. The circulars would be sent to the press and would undoubtedly be published. Addresses by health officers before medical societies and popular audiences, organisations of sanitary societies, the sanitary institutes of the department and the use in larger towns of our tuberculosis exhibit, would all prove powerful and effectual factors in the fight against the Great White Plague.

In this campaign of education the department will endeavor, acting with the health officers, health boards and public-spirited citisens, to organise anti-tuberculosis societies whose local influence and aid would be invaluable. For it must be steadily borne in mind that the greatest single factor in the winning of this battle is the constant and persistent dissemination of practical knowledge of the disease, its dangers and how to successfully combat it.

You have doubtless noted that the proposed stations serve not only as centers from which tuberculosis may be fought, but also as points where the antitoxin of the state laboratory may be obtained and, in some cases at least, bacteriological examinations made. The necessity for the promptest possible distribution of antitoxin is obvious and needs no comment. It may be said, however, that the high cost of commercial antitoxin seriously interferes with the sanitary control of diphtheria among the better classes. Diphtheria, which now stands fifth among diseases as a cause of mortality with 3,167 deaths yearly, will continue to maintain this position until sanitary authorities and the profession at large are as free to use it without restrictions as they now are to use ipecac or nux vomica.

In its bacteriological work the state laboratory labors under these two disadvantages: namely, physical inability to make speedy reports and lack of contact with the physician and health officer.

To obviate as far as possible the first difficulty, I have recently issued a notice to the effect that the reports upon the primary cultures from suspected cases of diphtheria and upon the Widal tests for typhoid fever be made by telegraph at the department's expense. This will effect a saving in some instances of twenty-four hours in placing these reports in your hands, and will relieve health officers from the burden of paying for such services out of their own pockets as has heretofore been too often the case. It is not unlikely that a further saving in time can be obtained in the future by the improvement in the details of the transportation of specimens, such as, for example, a provision for the incubation of cultures while en route.

To obviate the second difficulty, to a slight extent at least, I have had prepared a traveling bacteriological laboratory which will be sent with a trained laboratory diagnostician to those communities in which problems especially difficult of solution arise in connection with these diseases. Already the state laboratory has conducted one such field investigation in an extended and uncontrollable outbreak of mild diphtheria with, I believe, excellent results. But these provisions do not entirely overcome the difficulties or make the state routine bacteriological work the ideal method for the diagnosis of suspected diseases in the rural districts.

The local laboratory will always have its superior opportunities for the purely routine work. A movement has been started in this state by one of our most progressive health officers and his colleagues, and has resulted in the foundation of the Ontario County Laboratory at Canandaigua. The legislature passed a bill permitting the county authorities to pay the salary of a county bacteriologist. The building and equipment for the work was generously donated by a public-spirited woman, and the small fees obtained for the examinations of a private character which are made, pay at present for the incidental running expenses.

A plan of coöperation between this county laboratory and the state hygienic laboratory has been arranged by the directors of both institutions. Assistance is given by the state laboratory in connection with those technical procedures where operation in bulk tends toward economy. On the other hand, the county laboratory reports all the positive results of the examination of cultures, sputum and Widal tests to the State Laboratory. It would seem most desirable that this plan be introduced in other sections of the state.

Whenever such a laboratory is established, the state department will be prepared to make it a station of the department as outlined above, supplying a certain amount of apparatus, and aiding its efficient operation in every possible manner. I am of the opinion, however, in the development of this new work that provision should be made not only for a permissive coöperation between the county and the state laboratories, but that the county laboratory should be introduced into the public health service on somewhat the same legal basis as now applies to the relation between the local and the state health departments.

To this end I believe a bill should be introduced into the legislature during the coming session, permitting the establishment of hygienic laboratories by counties or groups of counties, and for a proper relation to the state hygienic laboratory and the state department of health. It will be seen at once what added strength the establishment of laboratory stations would bring in the fight against tuberculosis.

3. *State camps for consumptives.*—And now what care should the state give to the thousands of cases of incipient tuberculosis needing help?

If any care is to be exercised it is plain that it must be as simple and inexpensive as possible; otherwise the cost of caring for those people would be prohibitive. But we must always remember that we pay for them anyway. Whether we provide proper care or send them to almshouses and charity hospitals and thence to Potter's field, the expense is always ours. Remember, also, that every case removed to a suitable environment ceases to be a source of infection to his family, to his neighbors and the people in the streets.

I would advocate, therefore, the establishment of state camps for consumptives on state land. Plain board cabins should be erected, fashioned after sanitary plans. A little simple furniture should be provided. The inmates should be required to furnish, when able, all other furniture necessary. Such camps should have a resident physician and patients admitted only after proper and competent examination.

The results attained by such camps in other states and indeed in our own, have been most surprisingly gratifying and the percentage of cases can not be exceeded. I do not forget the very admirable Raybrook Hospital and the fine results reached there; but the state can not, it seems to me, commend itself to the policy of erecting such expensive buildings and demanding large allowances for maintenance. The number of consumptives seems to be too great to carry out such a plan, admirable though it may be. I believe that state camps instituted somewhat as outlined would produce the maximum of results at the minimum of expense.

4. *Local hospitals.*—There still remain for our consideration the chronic, the hopeless cases of tuberculosis. What shall be done with them? Should the state go as far as I have suggested, it would very probably deem it well to stop there, at least for the present.

Nor ought the state to do all of this work. The cities and towns of this state have responsibilities and duties which they too must recognise. It must be their part to care for the chronic cases of tuberculosis. Just how this should be done is a matter for determination by the local authorities. There is no time here to enter upon a discussion of methods.

Some such policy as the one I have tried to explain to you should be at once adopted by New York. I have only been able to present the bare outlines and many important matters have not even been alluded to. All that has been planned could not, of course, be done at once even if funds were available. But even with the little we have, we can begin this work and if nothing prevents I propose to make the first move on the 1st of January, 1908.

### THE PUBLIC SERVICE.

He succeeds best who serves best. For success is service and the greatest hero is the greatest helper. The difference that existed between the noblest emporer of the Romans and the meanest royal profligate who ever occupied a throne, is precisely the difference that obtains between great men and little men the world over. The one would advance the world; the other would advance himself. One would serve; the other would be served. The monuments made by printer's ink are but paper shafts that stand but for a moment; but he who writes himself upon the hearts of his fellow men, has made an epitaph that shall endure for generations.

With every true man his work is first, his fee second—very important, indeed, but still second. But in every walk in life there is a class ill-educated, cowardly, stupid. And with these just as certainly, the fee is first and the work second as with the noble, the work is first and the fee second. And this is no small distinction. It is, as Ruskin says, the whole distinction in a man: distinction between life and death *in* man, between heaven and hell *for* man.

"Society," said Burke, in his "Reflections on the Revolution in France," "Society is indeed a contract. It is a partnership in all science; a partnership in all art; a partnership in every virtue and in all perfection. As the ends of such a partnership can not be obtained in many generations, it becomes a partnership not only between those who are living, but between those who are dead, and those who are to be born." With but slight changes in phraseology this beautiful and impressive statement applies with great exactness to our organisation. It is for us with great opportunities for efficient service before us, to bear this partnership into which we have entered, constantly in mind. It lies with us to illustrate the meanness of an education which produces learned shirks or selfish skulkers or to illumine the perfection of a rounded culture with the radiant light of devotion to humanity. If difficult problems confront us, so much greater becomes the opportunity to solve them. If great obstacles are in the way, the greater glory to remove them. The watchword is service. This it is that crystallises our belief that enthusiasm and faith are the precursors of great deeds. As Dr. Van Dyke says, it impressively embodies our conception that the greatest success for a man, the only one at command, is to bring to his work a mighty heart. For it is more man that we need. Recent developments and discussion have driven us back upon the old, old truth—that only personality is the solution for the times—that all of the world's needs are embodied in its need for manhood.

About the plain and clear duties before us to perform we should have settled and fixed convictions. A man without convictions is a man of blubber. I do not say beliefs, opinions, views; all these are chaff in comparison. I say convictions so intertwined with his whole intellectual being, so coloring every thought, plan, purpose, labor, that they can no more be separated from them than his own existence can be separated from them. They should be seen and felt as powers wherever he goes; not because he is sounding a trumpet before him proclaiming their presence, announcing their glory, but because they are an inseparable part of his own personal character as the sun's brightness is of the sun's. So shall we labor that what come to us as seed shall go to the next generation as blossom and that which come to us as blossom may go to them as fruit. But in this fight in which we are justly engaged, the Empire State sends to us her message:

Have you heard it?

On the night of July 2, 1863, after a bloody battle a council of war was convened by the commanding general. The corps commanders present expressed their views. Slocum, being the ranking officer in the council, was the last to reply. He said: 'Stay and fight it out.' Slocum was not an orator, but no orator made a better speech. It was brief like Cesar's veni, vidi, vici, but it told the whole story. Stay and fight it out was the advice given by the council to General Meade, who was not satisfied with his position at Gettysburg. The army of the Potomac did stay and fight it out and the victory gained is the last comment that can be made.

That is the message of the Empire State—"Stay and fight it out." And on the unsullied surface of the scroll that shall record our advance during the coming year, first of all let us write across it in living letters of light the motto of this conference,

"STAY AND FIGHT IT OUT."

